# Patchy alopecia with pubic and axillary hair loss

**DOI:** 10.1016/j.jdcr.2025.03.035

**Published:** 2025-04-27

**Authors:** Zixun Zeng, Yinhao Ma, Gaiying Li, Yang Tang

**Affiliations:** Kunming Medical University First Affiliated Hospital, Plastic and Reconstructive Surgery, Kunming, China

**Keywords:** cicatricial alopecia, Graham-Little-Piccardi-Lassueur syndrome (GLPLS), lichen planopilaris, ophiasis, sisaipho

## Case report

**Fig 1:**
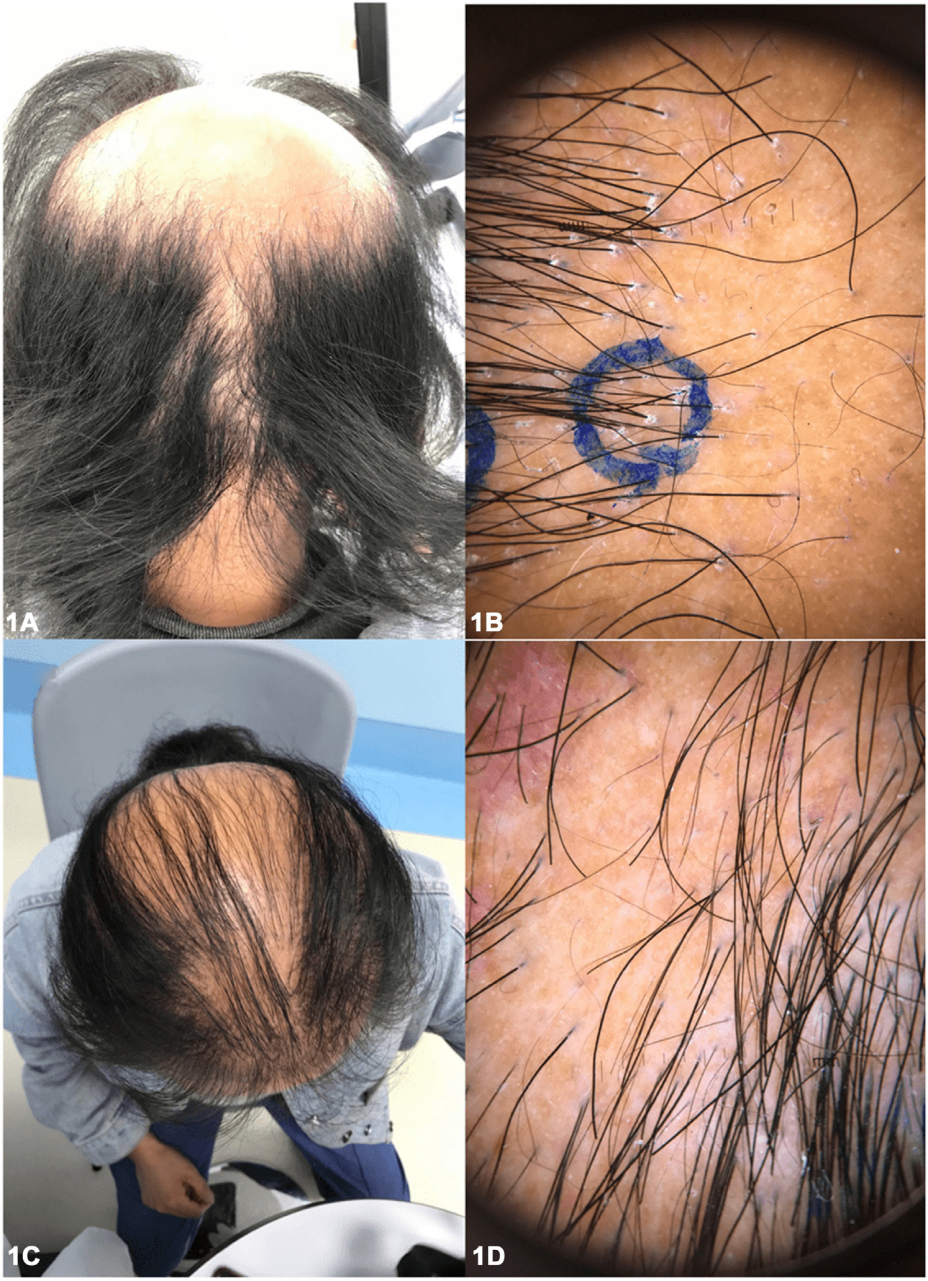

**Fig 2:**
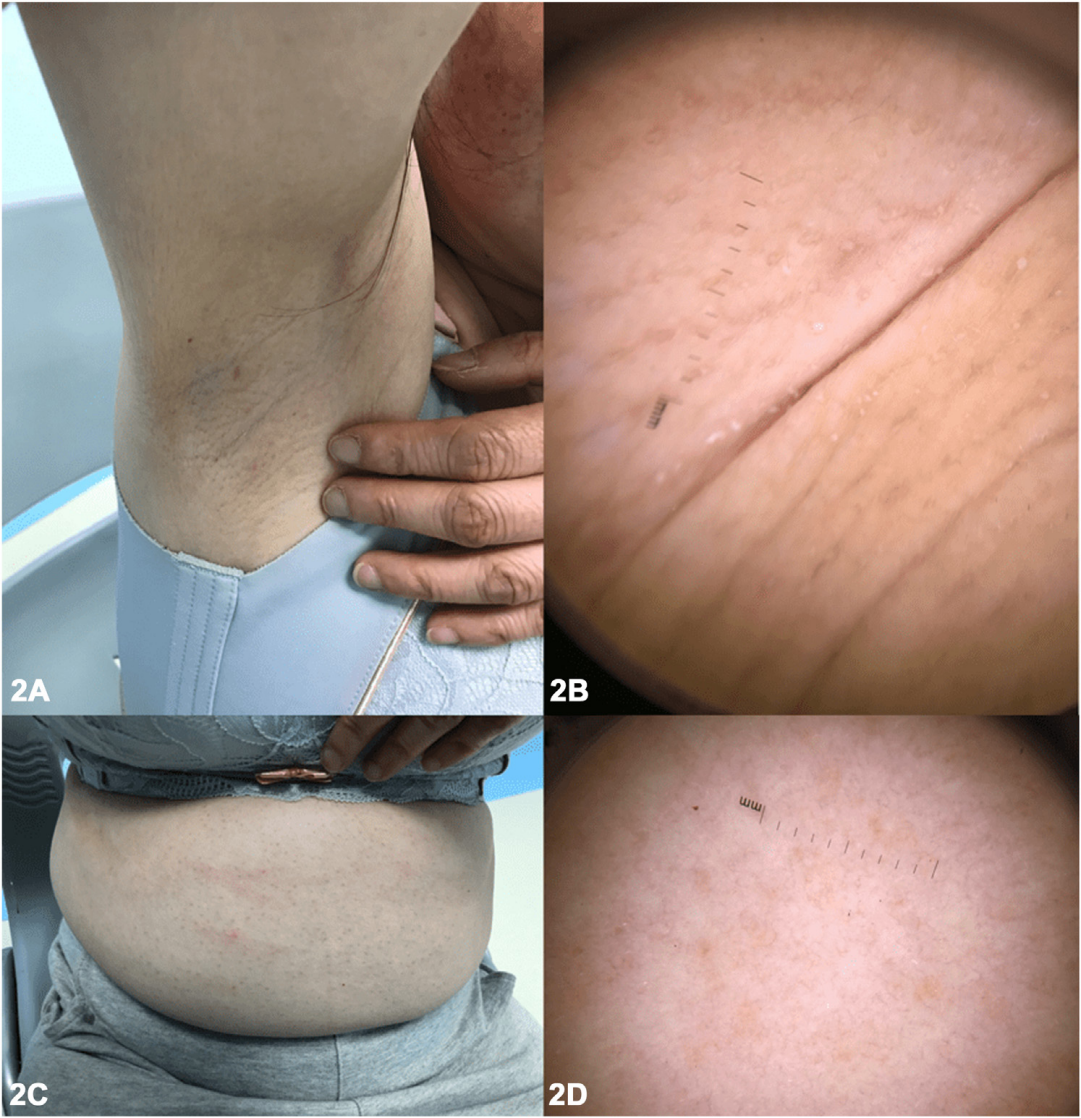

**Fig 3:**
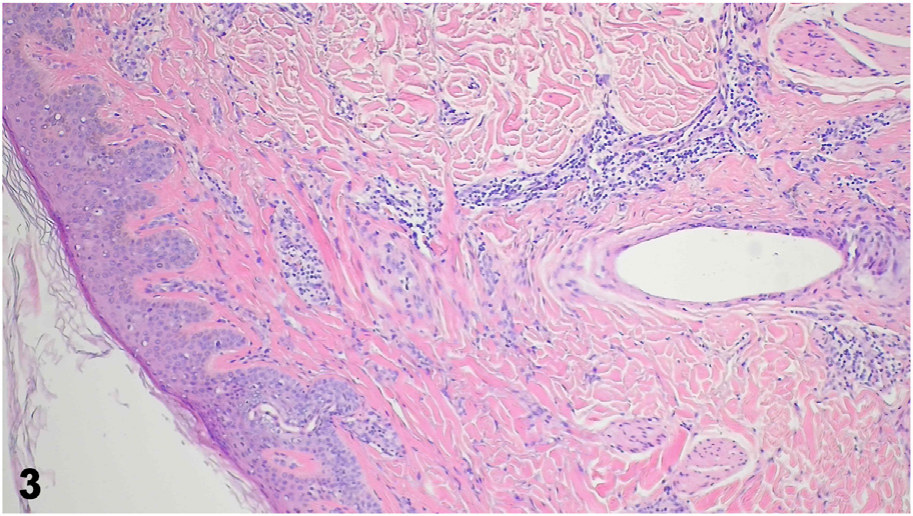

A 48-year-old female presented with a unique presentation of alopecia in a sisaipho-like pattern (Fig 1), accompanied by hair loss of the axillary and pubic regions, and multiple perifollicular keratotic papules on the abdomen (Fig 2). The patient had been experiencing these symptoms for nearly 10 years, along with severe pruritus and scaling around the hair follicles on her scalp. She reported no family history or any comorbid diseases. Laboratory tests, including autoimmune antibody panels, immunoglobulin levels, and thyroid function tests, were all within normal limits. Trichoscopy and punch biopsy of a scalp lesion were performed (Fig 3).

**Question 1: Which of the following is the most likely diagnosis?**

A. Alopecia areata (AA)
B. Androgenic alopecia (AGA)
C. Frontal fibrosing alopecia (FFA)
D. Graham-Little-Piccardi-Lassueur syndrome (GLPLS)
E. Traumatic scar

**Answers:**

A. Alopecia areata (AA) – Incorrect. AA is a nonscarring autoimmune hair loss condition with various subtypes, including patchy AA: localized hair loss in 1 or more areas; alopecia totalis (AT): complete hair loss on scalp; alopecia universalis (AU): complete hair loss of entire body; ophiasis: hair loss in a band-like pattern along the scalp; and sisaipho: the inverse pattern to ophiasis, affecting the crown and vertex region. In severe cases, AA can involve body hairs.
B. Androgenic alopecia (AGA) – Incorrect. Female AGA is a patterned, noncicatricial alopecia, typically presenting with an overall thinning of scalp hair, especially the frontal scalp and crown, while the frontal hairline remains intact.
C. Frontal fibrosing alopecia (FFA) – Incorrect. FFA, a variant of lichen planopilaris (LPP), primarily affects postmenopausal women and is characterized by a progressive, irreversible scarring hair loss that occurs in a band-like pattern along the frontal hairline. In some cases, the eyebrows may also be affected, while the axillary and pubic hairs typically remain intact.
D. Graham-Little-Piccardi-Lassueur syndrome (GLPLS) – Correct. GLPLS, a variant of LPP, is typically characterized by a distinctive triad of symptoms: progressive, irreversible hair loss on the scalp (Fig 1), nonscarring hair loss of armpit and/or pubis, and hyperkeratotic papules of trunk and/or extremities (Fig 2). Scalp punch biopsy showed decreased density of hair follicles and increased fibrosis with mild-to-moderate perifollicular and periadnexal lymphocytic infiltration (Fig 3).
E. Traumatic scar – Incorrect. Scalp trauma might result in geometric-looking alopecia of scalp. Usually, body hairs are unaffected. Additionally, the patient denied traumatic accident of scalp.

**Question 2: Which of the following is not a typical trichoscopic sign of GLPLS?**

A. Perifollicular scaling
B. Elongated, parallel-oriented blood vessels
C. Black dots
D. Violaceous area
E. Homogenous ivory-colored background

**Answers:**

A. Perifollicular scaling – Incorrect. The proximal segment of the hair shaft is encased in a tubular arrangement of scales, a condition also referred to as “tubular” or “collar-like” perifollicular hyperkeratosis. This excessive scaling of the outer root sheath of the hair follicle is associated with LPP and serves as a distinctive dermatological diagnostic indicator for this condition. GLPLS shares similar scalp trichoscopic signs with LPP.
B. Elongated, parallel-oriented blood vessels – Incorrect. In patients with LPP/GLPLS, more than 78.9% exhibit elongation of blood vessels adjacent to hair follicles.^1^
C. Black dots – Correct. Black dots represent the remnants of broken, pigmented hair shafts, indicating damage at the infundibulum level. This trichoscopic feature is commonly observed in conditions such as AA,^2^ trichotillomania, dissecting cellulitis, and can also appear incidentally in other dermatological disorders.^3^
D. Violaceous area – Incorrect. In patients with darker skin phototypes, melanin within the hair follicles may enter the catagen phase prematurely, leading to the formation of end-stage fibrotic tracts before complete involution. This process results in violaceous or gray areas surrounding empty hair follicle ostia, typically appearing in a targetoid pattern. It’s a typical trichoscopic sign for LPP/GLPLS.
E. Homogenous ivory-colored background – Incorrect. The absence of hair follicle openings in ivory-white or milky red areas is believed to be a residual effect of perifollicular fibrosis. This is a late-stage manifestation of LPP/GLPLS and can also be observed in other forms of folliculocentric cicatricial alopecia.

**Question 3: Which of the following is most likely the poor prognosticator for GLPLS?**

A. White reticular lesions of mucosa
B. Female gender
C. Family history of GLPLS
D. Comorbid disease
E. Chronic pruritus

**Answers:**

A. White reticular lesions of mucosa – Incorrect. Mucosal white reticular lesions are characteristics of typical lichen planus (LP) lesions. Cutaneous or mucosal LP appeared in up to 50% of patients with LPP, but it is rare in GLPLS. Among reported GLPLS cases, only 3 patients exhibited mucosal LP lesions. However, the extent of hair loss of them varied widely, ranging from single patch to complete alopecia (Supplementary Table I, available via Mendeley at https://data.mendeley.com/datasets/g3xyxzxm7c/1).
B. Female gender – Incorrect. Upon reviewing the existing literature, it appears that GLPLS primarily affects women. However, it is reported that male patients tend to experience this condition more severely, often resulting incomplete hair loss. Delayed medical attention or diagnosis may contribute to a more rapid progression and worsening of the condition (Supplementary Table II, available via Mendeley at https://data.mendeley.com/datasets/g3xyxzxm7c/1).
C. Family history of GLPLS – Incorrect. Nearly one-third of reported GLPLS cases claimed family history of GLPLS. However, the severity and progression of GLPLS varied significantly, even within families (Supplementary Table I, available via Mendeley at https://data.mendeley.com/datasets/g3xyxzxm7c/1).
D. Comorbid disease – Incorrect. The initial inflammatory factors in GLPLS remain unclear; proposed potential associations included Hepatitis B vaccination^4^ and androgen insensitivity.^5^ While not related to long-term prognosis, comorbid psychophysical diseases are common in female GLPLS patients, imposing significant burden and impacting the overall quality of life.
E. Chronic pruritus – Correct. More than half of the reported GLPLS patients experience pruritus, which may indicate the active stage of the disease. The prolonged duration of active phase appears to increase the risk of progression to more severe forms of GLPLS.

## Conflicts of interest

None disclosed.
